# Effects of exergaming with a resistance component versus traditional resistance training on sarcopenia in pre-frail and frail nursing home residents: a pilot randomized controlled trial

**DOI:** 10.1007/s41999-025-01294-w

**Published:** 2025-09-11

**Authors:** Tai Wa Liu, Bonny Y. M. Wong, Timothy T. T. Yam, William W. N. Tsang, Gary Tse, Queenie C. M. Kwan, Kevin Hung, John K. T. Chui, H. C. Wu, C. P. Leung, Janet Y. H. Wong

**Affiliations:** 1 School of Nursing and Health Sciences, Hong Kong Metropolitan University, Ho Man Tin, Hong Kong SAR China; 2 Exergaming Research Centre for Healthy Aging, Hong Kong Metropolitan University, Ho Man Tin, Hong Kong SAR China; 3 School of Science and Technology, Hong Kong Metropolitan University, Ho Man Tin, Hong Kong SAR China; 4 Department of Electrical and Electronic Engineering, The University of Hong Kong, Pokfulam, Hong Kong SAR China; 5 Hiu Kwong (Tak Yue) Nursing Centre, Yau Ma Tei, Hong Kong SAR China

**Keywords:** Exergaming, Resistance training, Sarcopenia, Frailty

## Abstract

**Purpose:**

To compare exergaming with a resistance component and traditional resistance training in terms of feasibility and the preliminary effects on sarcopenia, cognition, functional mobility, and frailty among pre-frail and frail nursing home residents.

**Methods:**

A two-arm pilot randomized controlled trial was conducted in three nursing homes. Thirty residents who met the criteria for pre-frailty or frailty and had low handgrip strength were randomized to participate in either exergaming with resistance (EGRG) or traditional resistance training (TRTG) for 12 weeks (twice-weekly, 40-min sessions). The primary outcomes included sarcopenia-related parameters (e.g., muscle mass, strength, lower extremity function, SARC-CalF). The secondary outcomes were cognition (HK-MoCA), functional mobility (TUG), and frailty (CFS-C). Assessments were performed at baseline and 6, 12, 16, and 24 weeks.

**Results:**

Thirty of 93 approached residents were enrolled and randomized (EGRG; *n* = 15, TRTG; *n* = 15), and 24 completed the study (80% retention). No significant group × time interaction was found for any outcome. Both groups showed significant improvements in handgrip strength, knee flexor/extensor strength, and the SPPB, TUG, and HK-MoCA scores from post-intervention to the 3-month follow-up. No significant changes were observed in the muscle mass, SARC-CalF, or CFS-C scores. Adherence was higher in the EGRG group (77.7%) than in the TRTG group (60.1%).

**Conclusion:**

Exergaming with a resistance component is feasible for pre-frail and frail nursing home residents and yields comparable improvements in muscle strength, physical function, and cognition to those achieved with traditional resistance training.

**Supplementary Information:**

The online version contains supplementary material available at 10.1007/s41999-025-01294-w.

## Key summary points

**Aim:**

To gain preliminary insights into the design, implementation, and effectiveness of an exergaming intervention with a resistance training component among pre-frail and frail nursing home residents.

**Findings:**

Both exergaming with a resistance training component and traditional resistance training led to significant improvements in muscle strength, lower extremity function, mobility, and cognition, but not in muscle mass or frailty status. Adherence was higher in the exergaming (vs. traditional) group, and the intervention was feasible and well-accepted.

**Message:**

Exergaming with a resistance training component is an engaging and practical alternative to traditional resistance training for improving sarcopenia, cognition, functional mobility, and frailty in nursing home residents at the pre-frail or frail stage.

**Supplementary Information:**

The online version contains supplementary material available at 10.1007/s41999-025-01294-w.

## Introduction

Sarcopenia, defined as the age-related loss of muscle mass and strength, is a major etiologic factor in frailty [1], a prevalent geriatric syndrome characterized by increases in vulnerability to stressors and the risk of adverse health outcomes [2]. Frailty arises from complex interactions between physiological decline and chronic conditions associated with aging [2]. It is especially common among nursing home residents, with an estimated prevalence ranging from approximately 40% to 52% depending on the population and assessment criteria used [3]. Importantly, frailty is a significant predictor of numerous negative health outcomes, including an increased risk of falls, worsening mobility, decreased ability to perform activities of daily living, and increased in all-cause hospitalization and all-cause mortality compared with non-frail older people [4]. Additionally, factors such as low serum vitamin D levels, anemia, and subclinical hyperthyroidism have been associated with an increased risk of frailty [5, 6].

Consistently, resistance training has been shown to improve muscle strength, functional performance, and cognitive functions and to reduce frailty-related parameters in older adults. A recent systematic review and meta-analysis found that resistance training interventions lasting at least 8 weeks produced small to large effects in terms of improved handgrip strength, lower limb strength, agility, gait speed, postural stability, functional performance, fat mass, and muscle mass [7]. Given the strong interrelationship between sarcopenia and frailty, interventions targeting sarcopenia-related parameters, including muscle strength and mass, are crucial for mitigating the progression of frailty and improving the quality of life of older people.

Cognitive impairment also plays a key role in the progression of frailty [8]. Non-frail older people outperform their frail counterparts in various cognitive domains, including processing speed, executive function, attention, working memory (including memory trace formation and maintenance and information retrieval and active manipulation), immediate memory, and delayed memory [9]. Cognitive function has also been shown to predict the incidence of frailty [10, 11]. In a cross-sectional study of older adults (age > 50 years), Lin et al. [12] found that poorer executive functioning was significantly associated with a higher risk of frailty. In a longitudinal study, Hu et al. [13] also demonstrated that cognitive functions, such as immediate and delayed recall and executive function, partially mediated the effect of frailty on the quality of life.

Exergaming has emerged as a promising intervention in the field of gerontechnology. It is highly adaptable, allowing the inclusion of various cognitive exercises, such as memory and reaction time training and physical exercises, such as balance training, to create individualized programs for rehabilitative training. Several commercial exergaming platforms are widely available, such as the Xbox system and Nintendo Wii. Ogawa et al. [14] systematically reviewed seven clinical trials, comprising five randomized controlled trials (RCTs) and two uncontrolled studies, and found that exergaming improved cognitive functions, including executive function, processing speed, and reaction time, in older adults. A recent RCT [15] demonstrated that a 12-week Kinect-based exergaming intervention significantly improved the global cognition in community-dwelling frail older adults. Participants in the Kinect group also showed significant improvements in verbal and working memory, whereas those in the combined exercise group did not.

Despite the well-documented benefits of resistance training and exergaming alone as promising interventions for older adults, it remains unclear whether exergaming with a resistance training component is acceptable and implementable for pre-frail and frail nursing home residents. Given the potential barriers to traditional exercise programs in these settings, including limited healthcare professional manpower, the aim of this pilot study is to gain preliminary insights into the design, implementation, and effectiveness of an exergaming intervention with a resistance training component for nursing home residents.

## Methods

### Study design

This two-arm pilot RCT with a 3-month follow-up was conducted in three nursing homes. The trial performance and reporting are compliant with the Standard Protocol Items: Recommendations for Interventional Trials (SPIRIT) statement [16] and CONSORT guidelines [17]. The trial is registered at https://clinicaltrials.gov (unique identifier: NCT05920577).

#### Project planning

During project planning, we collaborated closely with the management and staff of the three participating nursing homes. Initial meetings were held to discuss the challenges associated with the aging of nursing home residents. After our research team introduced the aim of the study and addressed logistical considerations (e.g., space and scheduling) and concerns regarding safety and the staff workload, the trial of an exergaming intervention with a resistance training component was confirmed.

#### Selection of exergaming and resistance training components

The exergaming and resistance training components were selected by our research panel, which comprised members with relevant exercise and exergaming experience, including a nurse, physiotherapist, and academic researcher in public health. The selection criteria for these components were as follows: (1) low-cost equipment; (2) safe and feasible activities in settings with limited space and staffing; (3) activities familiar to older adults in Hong Kong; (4) targeting of key domains relevant for sarcopenia management, including muscle strengthening, balance, and functional mobility; and (5) a resistance training component that can be administered easily by nursing home staff.

After a trial and discussion, the Nintendo Switch and the sport game series *Nintendo Switch Sports* were selected. Three exergaming components, namely badminton, tennis, and soccer, and a resistance training component involving weight cuffs were included in the intervention protocol. The badminton game required participants to perform repetitive arm swings and stepping movements, which promoted upper and lower limb coordination, muscle strength, and dynamic balance. The table tennis game emphasized upper limb movements, reaching, and weight shifting. In the soccer game, the participants engaged in simulated kicking and lower limb movements that involved repeated leg presses and lateral stepping, which targeted lower limb strength and postural control. Weight cuffs were used to add progressive resistance during the exergames, thus enhancing muscle activation in the shoulders, arms, and legs and promoting improved muscle strength, agility, and balance. The three selected exergames were also intended to stimulate cognitive processes, including attention, executive function, and reaction time, by requiring the players to quickly interpret visual cues, make rapid decisions, and coordinate complex motor responses. Details of the exergames are summarized in Table 1.

**Table 1:** Details of exergames

Exergames	Upper (U) or lower extremity exercise (L)	Left/right	Exercise duration (min)	Rest duration (min)	Total duration (min)
Soccer	L	Left	4	1	5
Badminton	U	Left	4	1	10
Soccer	L	Right	4	1	15
Tennis	U	Right	4	1	20
Soccer	L	Left	4	1	25
Badminton	U	Right	4	1	30
Soccer	L	Right	4	1	35
Tennis	U	Left	4	1	~ 40

Prior to the trial, the selected exergames and resistance training component were pre-piloted with a cognitively intact older adult. The older adult reported that the instructions and activities were easy to follow, the exergaming sessions were enjoyable and engaging, and the use of resistance equipment was comfortable. Minor suggestions included the use of clear visual and auditory cues. No significant difficulties were encountered during the pre-pilot test, thus supporting the feasibility and acceptability of the intervention protocol.

#### Training of research personnel

All the research personnel involved in the outcome assessments and intervention delivery participated in a structured training program. The aim of this training was to familiarize them with the assessment tools and intervention through hands-on demonstrations, supervised practice, use of the intervention equipment, and determination of the progression criteria. To ensure consistency, printed materials were provided, and return demonstrations were required. Only research personnel who achieved an acceptable level of reliability proceeded to data collection. The training also covered participant safety, particularly fall-preventing measures and emergency procedures.

#### Participants

To be eligible for this study, residents of the three nursing homes were required to (1) meet Fried’s criteria [4] for pre-frailty or frailty; (2) have low handgrip strength, defined as a handgrip strength < 28 kg for men and < 18 kg for women according to the Asian Working Group for Sarcopenia (AWGS), as these levels are considered to indicate a risk of sarcopenia [18]; (3) have adequate cognitive function (score ≥ 7 on the Chinese Abbreviated Mental Test) [19], and (4) be able to follow instructions. The exclusion criteria were involvement in other clinical trials, significant medical conditions, an inability to walk independently, or sensory impairments that could not be corrected.

Prior to recruitment, the research team organized a pre-study seminar with the nursing home residents and their relatives to explain the study details and invite participants. The seminar provided an opportunity to address questions and concerns, particularly about safety and insurance, and to encourage engagement with the study. Our research assistants then conducted the initial screenings and invited the nursing home residents to participate in the study.

#### Measurements

Assessments were conducted at baseline and at 6, 12 (end of the intervention), 16 (1-month follow-up), and 24 weeks (3-month follow-up). A 3-month follow-up was selected based on prior pilot RCTs demonstrating that this duration is sufficient to observe preliminary changes in outcomes while balancing participant retention [20].

#### Randomization and blinding

An off-site volunteer not involved in the other research procedures (recruitment, data collection, intervention) randomly allocated the participants to either the intervention group or the control group in a 1:1 ratio using the computer program Minimize [21]. Participants were stratified by age (60–69, 70–79, or 80–89 years), sex (male or female), and Fried frailty criteria (1 or 2, pre-frailty; 3 or more, frailty) [4]. Allocation concealment was achieved by keeping the randomization sequence secure and inaccessible to the research staff responsible for participant enrolment. Assessment, data entry, and data analysis were performed by another research assistant who was not involved in the randomization and intervention to maintain assessor blinding. All the participants were reminded not to disclose their group allocation to the assessor.

#### Interventions

##### Intervention group (i.e., exergaming with resistance component group, EGRG)

Participants in the EGRG engaged in twice-weekly sessions combining exergaming and resistance training with an approximate duration of 40 min for 12 weeks. The exergames were delivered via three Nintendo Switch sports games, namely badminton, tennis, and soccer, aiming to target both sarcopenia-related physical and cognitive health outcomes. Resistance was added through the use of cuff weights and was progressively increased based on perceived exertion (RPE). Details of the progression are summarized in the Supplementary Information (Appendix I).

##### Control group (i.e., traditional resistance training group, TRTG)

Participants in the TRTG performed traditional resistance training sessions with an approximate duration of 40 min twice-weekly for 12 weeks. The program included a warm-up and resistance exercises targeting the upper (handgrip, elbow flexion) and lower limbs (squats, single-leg stands, knee extensions) (Table 2). These exercises were intended to enhance muscle strength, endurance, and balance. Resistance was progressively increased according to the RPE and standardized progression criteria (Supplementary Information, Appendix II).

**Table 2:** Details of resistance exercises

	Resistance exercises	Details	Duration (min)	Total duration (min)
Lower limb	Warm up on ergometer	At a comfortable speed	5	5
	Squat (sit-to-stand)	6 reps (6 s). × 3 sets (30 s rest between set)	5	10
	Single-leg standing (L/R)	Hold max 1 min with light support for 3 times, × 3 sets (20 s rest between set)	6	16
	Knee extension (L/R)	10 reps, × 3 sets (hold 3–5 s) (30 s rest between set)	6	22
Upper limb	Warm up on ergometer	At a comfortable speed	5	27
	Hand grip (L/R)	Theraweb or putty can be used 10 reps, × 3 sets (hold 3–5 s) (30 s rest between set)	6	33
	Elbow flexion (in sitting) (L/R)	10 reps, × 3 sets (hold 3–5 s) (30 s rest between set)	6	39

#### Outcome measures

The primary outcomes were sarcopenia-related parameters according to the operational definition of sarcopenia revised by the European Working Group on Sarcopenia in Older People [1]. The secondary outcomes were cognition, functional mobility, and frailty.

##### Primary outcome: sarcopenia-related parameters

Muscle quantity. This parameter was assessed using the Appendicular Skeletal Muscle Mass Index (ASMI), measured with a bioelectrical impedance analysis (BIA) device. ASMI is calculated by dividing the appendicular skeletal muscle mass by the square of the participant’s height according to the guidelines of the AWGS and the European Working Group on Sarcopenia in Older People (EWGSOP) [1, 18]. This normalization accounts for differences in body size [1, 18].

Muscle strength. Handgrip strength was measured bilaterally using a Jamar Grip Dynamometer (kg)(Sammons Preston Rolyan, Bolingbrook, Illionis, USA). Each participant sat upright with their dominant arm parallel to the body, elbow flexed at 90°, and forearm and wrist in a neutral position. They performed maximal isometric contractions for 3 s per trial and were verbally encouraged using the standard phrase “Push against my resistance as hard as you can.” Three trials were conducted, with rest intervals of at least 1 min between trials to minimize fatigue. The mean peak value of the three trials was used for data analysis. Handgrip strength measurement with a handheld dynamometer was shown to have good to excellent (intraclass correlation coefficient [ICC] > 0.95) [22] and excellent (ICC 0.90–0.98) test–retest reliability among nursing home residents and community-dwelling older adults [23], respectively. Knee flexor and extensor strength were measured using a handheld dynamometer (Jamar) to assess the maximum voluntary isometric contractions in accordance with a standardized assessment protocol [24]. Each participant was seated with their hips and knees flexed at approximately 90 degrees. The dynamometer was positioned against the lower leg just above the ankle. Participants were instructed to push (for extensors) or pull (for flexors) against the dynamometer with maximal effort for 3 s. Three trials were performed for each muscle group, with rest intervals of at least 1 min between trials. The peak force from each trial was recorded, and the mean of the three trials was used for analysis.

Lower extremity functions. The Short Physical Performance Battery (SPPB) was used to assess lower extremity function in older adults [25]. This test comprises three subtests: walking speed (4-m walk), repeated chair stands, and standing balance. Each subtest is scored from 0 (unable to complete) to 4 (best performance), yielding a total score ranging from 0 to 12, with higher scores indicating better function. For the chair stand test, the participant sat with their arms crossed, then stood up and sat down five times consecutively. The 4-m walk test measured the usual gait speed over the middle 4 m of a 6-m path. The standing balance test assessed the ability to hold three positions for up to 10 s each: feet together, semi-tandem stance (side of heel touching big toe), and tandem stance (heel in front of toes). Although the SPPB has demonstrated variable interrater reliability (ICC = 0.33–0.84), it is widely used to evaluate physical performance and predict functional decline in older adults [26].

Sarcopenia. Strength, Assistance with walking, Rise from a chair, Climb stairs and (Calf) Falls (SARC-CalF) is a well-known screening tool for sarcopenia; it consists of five items with scores ranging from 0 to 2 per item [27]. It was found to exhibit high inter-rater (ICC: 0.93 (95% confidence interval [CI]: 0.82–0.97)) and test–retest reliability (0.90 (0.78–0.95)) [28]. A total score of ≤ 11 was considered to indicate a risk of sarcopenia.

##### Secondary outcomes

Cognition. The Montreal Cognitive Assessment (MoCA) [29] is a clinical measure of various cognitive domains significantly affected by age-related cognitive decline, such as short-term memory recall and visuospatial abilities. The Hong Kong version (HK-MoCA) was reported to have good to excellent test–retest reliability (ICC = 0.82–0.92) among people with mild neurocognitive disorders [30].

Functional mobility. The Timed Up and Go Test (TUG) was used to measure functional mobility. The subject was asked to rise from a chair without armrests, walk 3 m, turn around, and return to the chair to sit down as quickly as possible during the test. The average completion time of three trials was calculated. The TUG test was shown to have excellent test–retest reliability (ICC ≥ 0.93) among community-dwelling older people [31].

Frailty. The level of frailty was measured using the Chinese version of the Clinical Frailty Scale (CFS-C) [32]. It contains nine items and was found to be associated with mortality, comorbidity, cognition, falls, and functioning. CFS-C has good criterion validity (strong correlation with the Frailty Index based on Comprehensive Geriatric Assessment (Kendall’s tau = 0.63) and good inter-rater reliability with a strong correlation (Weighted kappa = 0.60; Kendall’s tau = 0.67) in Chinese older adults.

#### Data analysis and sample size

All the data were processed using SPSS 23.0 (IBM, Armonk, NY, USA). A significance level of p < 0.05 (two-sided) was adopted. Descriptive statistics are used to summarize the participants’ characteristics. The attendance rate was calculated for each participant as the percentage of attended sessions out of the total number of scheduled sessions. Participants were categorized as having high (attendance rate ≥ 75%) or low (attendance rate ≤ 75%) adherence for subgroup analyses conducted post-intervention and at the 3-month follow-up. Due to the small subgroup sample sizes, descriptive statistics were used rather than formal hypothesis testing to identify potential trends in subgroup analysis. Between-group baseline comparisons were conducted using the Mann–Whitney *U* test, chi-square test, or Fisher’s exact test, as appropriate. Linear mixed-effects models (LMM) were used to measure changes over time in the variables of interest between the two study arms (group effect) from baseline to the four assessment time points (time effect). The LMM approach accounts for intra-correlated repeated measures data and automatically adjusts for missing data caused by dropouts under the assumption that they are missing at random. The models were adjusted for potential confounders, including sociodemographic variables and outcome parameters, that differed between the groups at baseline. A post hoc analysis with Bonferroni adjustments was performed if significant interactions and/or significant time effects were observed. The intention-to-treat principle was adopted for data analysis.

The total sample size of this RCT was 30. It was based on the recommendation that a minimum of 12 participants per group would be needed to estimate key parameters with an assumed 25% attrition rate (12 × 1.25 × 2 groups) [33].

### Ethics considerations

Ethics approval for this study was received from the Research Ethics Committee of the Hong Kong Metropolitan University, and the study was conducted according to the Declaration of Helsinki [34]. The details were explained to all the participants, and written consent was obtained before the study commenced.

## Results

**Fig. 1:**
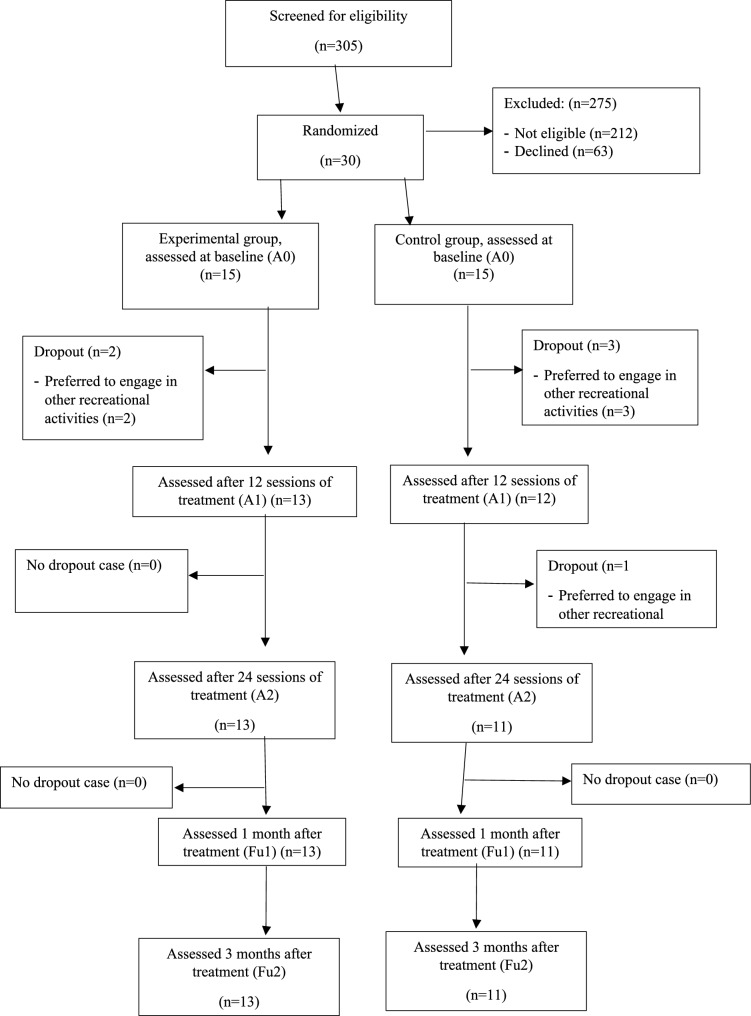
Flow of the study

Of the 93 nursing home residents approached between April 2024 and April 2025, 30 met the eligibility criteria and consented to participate (Fig. 1). All 30 participants were randomized to the EGRG or TRTG at a 1:1 ratio, and 24 (EGRG, *n* = 13; TRTG, *n* = 11) completed the 12-week intervention and follow-up assessments (retention rate = 80%). The participants’ baseline characteristics are summarized in Table 3. No significant between-group differences in sociodemographic data and outcome parameters were found at baseline. No adverse events related to the interventions were reported.

**Table 3:** Baseline characteristics of participants (*n* = 30)

	TRTG (*n* = 15)	EGRG (*n* = 15)	*U*, *χ*^2^ (*p* value)
Age in years, mean ± SD^a^	76.13 ± 8.16	75.80 ± 6.99	111.0 (0.950)
Sex, *n* (%)^b^			0.133 (0.715)
Women	7 (53.3)	8 (46.7)	
Men	8 (46.7)	7 (53.3)	
BMI, mean ± SD^a^	24.02 ± 5.88	22.49 ± 4.31	95.0 (0.468)
Frailty Phenotype, *n* (%)			(0.674)
Pre-Frail	12 (80)	12 (80)	
Frail	3 (20)	3 (20)	
ASMI (score, higher is better), mean ± SD^a^	6.92 ± 0.90	7.07 ± 1.14	99.0 (0.575)
Muscle strength (kg, higher is better), mean ± SD^a^			
Handgrip (kg)^a^	13.61 ± 7.67	14.74 ± 7.90	102.0 (0.663)
Knee flexors (kg)^a^	7.99 ± 2.61	8.30 ± 3.13	110.5 (0.934)
Knee extensors (kg)^a^	8.38 ± 1.84	8.54 ± 3.20	98.0 (0.548)
SPPB (score 0–12, higher is better), mean ± SD^a^	6.60 ± 1.60	7.0 ± 1.60	97.0 (0.513)
SARC-CalF (score 0–20, lower is better), mean ± SD^a^	7.73 ± 5.24	8.60 ± 5.44	95.0 (0.427)
HK-MoCA (score 0–30, higher is better), mean ± SD^a^	20.87 ± 0.99	21.0 ± 1.25	100.0 (0.588)
TUG (completion time, s, faster is better), mean ± SD^a^	16.19 ± 4.61	16.12 ± 4.40	109.50 (0.901)
CFS-C (score 1–9, lower is better), mean ± SD^a^	4.07 ± 0.80	3.80 ± 1.01	99.50 (0.563)

^a^Mann–Whitney *U* test

^b^Chi-square test

*BMI* body mass index, *ASMI* Appendicular Skeletal Muscle Mass Index, *SPPB* Short Physical Performance Battery, *SARC-CalF* Strength, Assistance with Walking, Rise from a Chair, Climb Stairs and Falls, *HK-MoCA* Hong Kong version-Montreal Cognitive Assessment, *TUG* Timed Up and Go Test, *CFS-C* Chinese version of Clinical Frailty Scale, *EGRG* exergaming with resistance component group, *TRTG* traditional resistance training group

### Adherence

The mean attendance rates in the EGRG and TRTG over the 12-week intervention period were 77.7% and 60.1%, respectively. The average attendance rates for EGRG and TRTG completers were 41.7% and 79.6%, respectively, while those for dropouts in the EGRG and TRTG were 39.6% and 25.0%, respectively. Within both groups, participants in the high adherence subgroup tended to show greater improvements in all the outcome measures at the 3-month follow-up, except for the CFS-C score in the EGRG (Supplementary Information, Appendix III).

### Acceptability and feedback

The informal feedback received from the participants indicated that the exergaming sessions with a resistance training component were enjoyable and provided a novel form of engagement. Nursing home staff also reported that the intervention was feasible to implement within the institutions’ routines as it could be conducted at any time and required limited manpower and space.

### Efficacy outcomes

No significant group-by-time interaction effect was identified with respect to the ASMI, handgrip strength, knee flexor strength, knee extensor strength, SPPB score, SAR-Calf score, TUG completion time, HK-MoCA score, or CFS-C score (Table 4). Compared with the baseline values, both the EGRG and TRTG demonstrated significant improvements in the handgrip strength, knee flexor strength, knee extensor strength, SPPB score, TUG completion time, and HK-MoCA score from post-intervention to the 3-month follow-up.

**Table 4:** Effects of variables of interests

Outcome	Group	Baseline mean (SE)	Week 6 mean (SE)	Week 12 mean (SE)	Week 16 mean (SE)	Week 24 mean (SE)	Interpretation summary
ASMI (score, higher is better)	TRTG	6.917 (0.268)	7.005 (0.296)	7.027 (0.289)	7.016 (0.288)	7.023 (0.286)	• No significant group, time or group × time effects
	EGRG	7.071 (0.268)	7.143 (0.295)	7.164 (0.288)	7.151 (0.288)	7.140 (0.286)	
Handgrip strength (kg, higher is better)	TRTG	13.614 (3.153)	13.835 (3.153)	14.293 (3.151)	14.279 (3.151)	14.292 (3.151)	• No significant group effect or group × time effects • Significant time effect from mid-intervention (week 6) up to 3-month follow-up
	EGRG	14.740 (3.153)	14.963 (3.153)	15.255 (3.151)	15.232 (3.151)	15.268 (3.151)	
Knee flexors strength (kg, higher is better)	TRTG	7.989 (0.758)	8.115 (0.759)	8.350 (0.759)	8.320 (0.759)	8.344 (0.759)	• No significant group effect or group × time effects • Significant time effect from mid-intervention (week 6) up to 3-month follow-up
	EGRG	8.304 (0.758)	8.352 (0.759)	8.579 (0.759)	8.563 (0.759)	8.639 (0.759)	
Knee extensors strength (kg, higher is better)	TRTG	8.376 (0.700)	8.721 (0.703)	9.314 (0.704)	9.337 (0.704)	9.336 (0.704)	• No significant group effect or group × time effects • Significant time effect from mid-intervention (week 6) up to 3-month follow-up
	EGRG	8.538 (0.70)	8.631 (0.702)	9.152 (0.702)	9.162 (0.702)	9.158 (0.702)	
SPBB (score 0–12, higher is better)	TRTG	6.60 (0.441)	6.763 (0.445)	7.320 (0.446)	7.320 (0.446)	7.320 (0.446)	• No significant group effect or group × time effects • Significant time effect from mid-intervention (week 6) up to 3-month follow-up
	EGRG	7.0 (0.441)	7.305 (0.443)	7.613 (0.443)	7.613 (0.443)	7.536 (0.443)	
SARC-CalF (score 0–20, lower is better)	TRTG	7.733 (1.690)	7.713 (1.625)	7.494 (1.582)	7.578 (1.573)	7.577 (1.572)	• No significant group, time or group × time effects
	EGRG	8.60 (1.690)	8.637 (1.624)	8.503 (1.582)	8.431 (1.572)	8.432 (1.572)	
TUG (completion, s, faster is better)	TRTG	16.192 (1.267)	16.013 (1.280)	15.301 (1.290)	15.262 (1.256)	15.308 (1.253)	• No significant group effect or group × time effects • Significant time effect from mid-intervention (week 6) up to 3-month follow-up
	EGRG	16.123 (1.267)	16.072 (1.279)	15.578 (1.289)	15.610 (1.254)	15.672 (1.252)	
HK-MoCA (score 0–30, higher is better)	TRTG	20.867 (0.294)	20.952 (0.308)	21.186 (0.381)	21.281 (0.375)	21.198 (0.366)	• No significant group effect or group × time effects • Significant time effect from mid-intervention up to 3-month follow-up
	EGRG	21.0 (0.294)	21.228 (0.307)	21.639 (0.376)	21.637 (0.370)	21.632 (0.361)	
CFS-C (score 1–9, lower is better)	TRTG	4.067 (0.234)	4.067 (0.236)	3.975 (0.237)	4.066 (0.237)	4.156 (0.237)	• No significant group, time or group × time effects
	EGRG	3.80 (0.234)	3.802 (0.235)	3.648 (0.235)	3.648 (0.235)	3.802 (0.235)	

*SE* standard error, *ASMI* Appendicular Skeletal Muscle Mass Index, *SPPB* Short Physical Performance Battery, *SARC-CalF* Strength, Assistance with Walking, Rise from a Chair, Climb Stairs and Falls, *HK-MoCA* Hong Kong version-Montreal Cognitive Assessment, *TUG* Timed Up and Go Test, *CFS-C* Chinese version of Clinical Frailty Scale, *EGRG* exergaming with resistance component group, *TRTG* traditional resistance training group

## Discussion

This study evaluated the feasibility and effectiveness of an exergaming intervention involving a commercially available gaming console and a resistance training component for pre-frail or frail nursing home residents with respect to improvements in sarcopenia, cognition, and functional mobility. We found that the effects of exergaming with a resistance component were comparable to the well-proven effects of traditional resistance training alone, with observed carry-over effects on muscle strength, lower extremity function, functional mobility, and cognitive function. Our pilot study also highlighted several key issues. First, engaging and motivating interventions were required to encourage adherence; here, the combination of exergaming with resistance training was feasible for and acceptable among nursing home residents. Second, standardized research personnel training, clear protocols, and effective communication with nursing home staff, residents, and their relatives were essential to minimize measurement variability and streamline project implementation. These insights can inform the design and delivery of future exergaming-related interventions for nursing home residents.

Previous studies have reported that exergaming can improve exercise adherence. Anderson-Hanley et al. [35] explored the relationship between exercise behavior, self-regulation, and executive control in the post-intervention period of an RCT comparing exergaming (a virtual reality stationary bike) with traditional exercise. The authors found that older adults with cognitive decline exercised more frequently with (vs. without) exergaming, possibly because they were motivated by the potential of gaining cognitive benefits unique to exergaming [35]. This finding suggests that exergaming may especially engage people with cognitive concerns and thus might improve adherence in this subgroup. Ho et al. [36] systematically reviewed five RCTs that compared the effects of exergaming with active control (conventional physical exercises without game components) and inactive control (e.g., waitlist control) on frailty in older adults. The authors revealed no significant difference between the conditions in terms of frailty-related health parameters but observed a slightly higher adherence rate in the exergaming group (87.3%–87.7%) than the comparison group (81.1%–85.4%). In the current study, the adherence rate was higher in the EGRG than in the TRTG. This finding echoes the study by Ho et al. [36], which showed comparable effects between exergaming and traditional physical exercise; thus, clinicians are recommended to consider using exergaming with or without traditional physical exercise due to its engaging and motivating features. Importantly, a favorable pattern of improvement was observed among the participants with a high attendance rate in both the EGRT and TRTG. This finding suggests that higher adherence, regardless of intervention type, may be associated with more favorable outcomes. However, these trends warrant further investigation in larger, adequately powered studies. The findings of this pilot RCT add to a growing body of evidence regarding the effects of traditional resistance training and exergaming in pre-frail or frail older people, particularly those residing in nursing homes. Our findings are consistent with previous literature stating that both resistance training [37–39] and exergaming [40] can improve muscle strength, lower extremity function, functional mobility, and cognitive function. However, neither intervention produced significant within-group improvements in muscle mass, which may be explained by two factors.

First, our participants were pre-frail or frail older people who exhibited anabolic resistance, a blunted muscle protein synthesis response to exercise that requires longer or more intensive interventions to achieve measurable muscle mass gains [40]. Second, the BIA device used in the present study may not have been sensitive enough to detect small changes in muscle mass in this population.

The lack of a significant improvement in sarcopenia status according to SARC-CalF screening may reflect the inherent limitations of this measurement tool. SARC-CalF combines subjective questionnaire items with calf circumference measurement. The questionnaire focuses on difficulties with various functional daily activities, such as lifting or carrying 10 pounds and climbing stairs, which may not capture subtle improvements in muscle strength, lower extremity functions, or functional mobility. In addition, anabolic resistance may have limited increases in the participants’ calf circumference [41], which would have further reduced the tool’s sensitivity. These findings suggest that functional improvements, including lower extremity functions and functional mobility, can be achieved in pre-frail or frail older people even in the absence of substantial gains in muscle mass.

Despite improvements in physical and cognitive functions, our study did not observe a significant change in the frailty status as measured by the CFS-C. There are two possible explanations for this outcome. First, the CFS is a clinician-rated tool that relies heavily on overall clinical impressions. It is sensitive to significant changes in health status but may not detect subtle improvements, especially if these changes do not translate into a clear shift in the overall clinical presentation of frailty. This limitation has been noted in previous studies in which the CFS was not sufficiently responsive to small, yet meaningful, improvements in physical or cognitive domains [42]. Second, frailty is a complex clinical syndrome influenced by a range of factors beyond muscle strength and mobility, such as mood. Even when our resistance training and exergaming improved specific domains, the overall frailty status might have remained unchanged unless there were broader, clinically significant improvements across multiple domains [43].

Prior to the start of the study, we searched PubMed and found no published clinical trials or systematic reviews directly comparing exergaming with resistance components and traditional resistance training with the aim of improving sarcopenia among older people, particularly pre-frail or frail nursing home residents. Some RCTs have reported comparable improvements in health outcomes between exergaming and traditional resistance training, aligning with our finding of significant within-group effects but not between-group effects with respect to one intervention vs. the other [44, 45]. For example, Gschwind et al. [44] found that both exergaming and traditional exercise improved balance and reduced the risk of falls in community-dwelling older adults but found no significant differences between the groups.

In this study, we used cuff weights as the resistance training component during exergaming sessions to enhance muscle loading and simulate traditional resistance exercise. While this was an easy-to-use and low-cost means of adding resistance, previous studies have used a range of resistance modalities, such as elastic bands, weight machines, and bodyweight exercises, when integrating resistance training into exercise programs for older adults [37, 46]. These resistance modalities may vary in their degree of effectiveness in improving muscle strength, physical performance, and even adherence, depending on the population and setting [47]. This finding highlights an important advantage of exergaming platforms: they can be individualized or adapted to incorporate a variety of resistance training components, thus allowing the interventions to be customized to the needs, abilities, and preferences of older adults.

## Strengths and limitations

This study offered a combined intervention that could be incorporated into the routine care provided to nursing home residents without incurring significant costs. Furthermore, the high completion and adherence rates observed in this study support the feasibility of exergaming with a resistance training component in this population. However, several limitations should be acknowledged. First, a formal sample size calculation was not performed due to the exploratory nature of the trial; this may have limited the statistical power to detect small but clinically meaningful effects. However, our sample size of 15 participants per group met a prior recommendation (minimum of 12 participants per group) for estimating the key parameters of our study [33]. Second, the study was conducted using a relatively homogenous sample, which may have limited the generalizability of the findings and precluded us from drawing definitive conclusions about comparative effectiveness. Lastly, the lack of participant blinding might have introduced performance bias, although assessor blinding was maintained. In summary, our findings suggest that exergaming can enhance engagement and adherence in older adults, supporting its potential role in geriatric care. Future research with larger and longer-term studies is needed to clarify the comparative effectiveness of various resistance modalities within exergaming interventions, and to identify optimal strategies for managing sarcopenia and frailty.

## Supplementary Information

Below is the link to the electronic supplementary material.

Supplementary file1 (DOCX 23 KB)
