# Supplementary material for: Effects of exergaming with a resistance component versus traditional resistance training on sarcopenia in pre-frail and frail nursing home residents: a pilot randomized controlled trial
Source: Eur Geriatr Med. 2025 Sep 11;17(2):697–709. doi: 10.1007/s41999-025-01294-w (PMC13109284; doi:10.1007/s41999-025-01294-w)
Supplement: Supplementary file 1 — Supplementary file1 (DOCX 23 KB) [file 41999_2025_1294_MOESM1_ESM.docx]

**Appendix I Progression of exergames**

| Week | Progression |
| --- | --- |
| 1 & 2 | No resistance, RPE 11 -12 |
| 3 – 5 | Add light cuff weight, RPE 12 – 13 |
| 6 – 8 | Increase 10% light cuff weight if RPE is lower than 12; RPE 13 – 14 |
| 9 – 12 | Increase 10% light cuff weight if RPE is less than 12; RPE 13 - 15 |

RPE, rate of perceived exertion

**Appendix II Progression of resistance training**

|  | Week 1-2 | Week 3-5 | Week 6-8 | Week 9-12 |
| --- | --- | --- | --- | --- |
| Lower limb | | | | |
| Warm up on ergometer | Remains the same repetition 9-11 | | | |
| Squat (sit-to-stand) | RPE 11-12  No additional weight | RPE 12-13  6 🡪 7 repetitions | RPE 13-14  Keep at 7 repetitions | RPE 13-15  7 🡪 8 repetitions |
| Single-leg standing  (L/R) |  | RPE 12-13  3 🡪 4 times | RPE 13-14  4 🡪 5 times | RPE 13-15  5 🡪 6 times |
| Knee extension  (L/R) |  | RPE 12-13  Add light cuff weight | RPE 13-14  Increase ~10% weight if participant can perform 2 additional reps per set | RPE 13-15  Increase ~10% weight if participant can perform 2 additional reps per set |
| **Upper limb** | | | | |
| Warm up on ergometer | Remains the same RPE 9-11 | | | |
| Hand grip  (L/R) | RPE 11-12  Yellow theraweb | RPE 12-13  Yellow theraweb | RPE 13-14  Red theraweb | RPE 13-15  Red or blue theraweb |
| Elbow flexion (in sitting)  (L/R) | RPE 11-12  Light weight | RPE 12-13  Increase ~10% weight if participant can perform 2 additional reps per set | RPE 13-14  Increase ~10% weight if participant can perform 2 additional reps per set | RPE 13-15  Increase ~10% weight if participant can perform 2 additional reps per set |

RPE, rate of perceived exertion

**Appendix III Descriptive statistics for outcome measures by adherence subgroup**

|  | |  | TRTG | |  | EGRG | |  |
| --- | --- | --- | --- | --- | --- | --- | --- | --- |
| Outcome measure | | Time point, mean±SD | High adherence group | Low adherence group | Mean difference (Post-intervention-baseline; 3-month follow up-baseline) | High adherence group | Low adherence group | Mean difference (SD); Post-intervention-baseline; 3-month follow up-baseline |
| ASMI (score, higher is better) | | Baseline | 7.18±1.03 | 6.53±0.53 |  | 7.45±1.09 | 6.32±0.94 |  |
|  | | Post-intervention | 7.37±1.09 | 6.57±0.65 | 0.19 (0.19); -0.10 (0.06) | 7.69±1.21 | 6.24±0.91 | 0.20 (0.14); -0.08 (0.07) |
|  | | 3-month follow-up | 7.41±1.15 | 6.55±0.63 | 0.22 (0.08); -0.12 (0.04) | 7.65±1.20 | 6.24±0.93 | 0.16 (0.09); -0.08 (0.06) |
| Muscle strength (kg, higher is better) | |  |  |  |  |  |  |  |
|  | Handgrip (kg) | Baseline | 12.73±6.87 | 14.94±9.25 |  | 15.62±7.79 | 12.99±8.74 |  |
|  |  | Post-intervention | 13.68±7.46 | 17.31±8.87 | 0.87 (0.16); 0.10 (0.11) | 16.80±8.53 | 12.99±8.75 | 0.83 (0.38); 0.0 (0.10) |
|  |  | 3-month follow-up | 13.67±7.39 | 17.33±8.79 | 0.86 (0.23); 0.12 (0.33) | 16.82±8.64 | 12.99±8.77 | 0.85 (0.32); 0.01 (0.16) |
|  | Knee flexors (kg) | Baseline | 8.02±1.97 | 7.95±3.59 |  | 8.87±3.67 | 7.17±1.30 |  |
|  |  | Post-intervention | 8.44±2.28 | 8.01±3.94 | 0.47 (0.24); 0.04 (0.09) | 9.23±3.94 | 7.13±1.25 | 0.47 (0.11); -0.04 (0.08) |
|  |  | 3-month follow-up | 8.41±2.27 | 8.08±3.96 | 0.44 (0.26); 0.10 (0.11) | 9.21±3.86 | 7.31±1.26 | 0.45 (0.09); 0.15 (0.45) |
|  | Knee extensors (kg) | Baseline | 8.36±1.19 | 8.40±2.69 |  | 9.46±3.55 | 6.69±1.02 |  |
|  |  | Post-intervention | 9.42±0.82 | 9.25±3.75 | 1.22 (0.47); 0.12 (0.04) | 9.96±3.64 | 6.73±1.05 | 0.98 (0.19); 0.05 (0.04) |
|  |  | 3-month follow-up | 9.47±0.85 | 9.21±3.75 | 1.99 (1.27); 0.07 (0.09) | 9.96±3.56 | 6.74±1.04 | 0.99 (0.12); 0.05 (0.03) |
| SPPB (score 0-12, higher is better) | | Baseline | 6.22±1.56 | 7.17±1.60 |  | 7.10±1.79 | 6.80±1.30 |  |
|  | | Post-intervention | 7.38±2.0 | 7.67±2.08 | 0.88 (0.83); 0.33 (0.58) | 7.75±2.32 | 7.20±1.30 | 0.75 (0.46); 0.40 (0.55) |
|  | | 3-month follow-up | 7.38±2.0 | 7.67±2.08 | 0.88 (0.83); 0.33 (0.58) | 7.63±2.33 | 7.20±1.30 | 0.63 (0.52); 0.40 (0.55) |
| SARC-CalF (score 0-20, lower is better), mean±SD* | | Baseline | 7.56±5.41 | 8.0±5.48 |  | 7.30±5.56 | 11.20±4.60 |  |
|  | | Post-intervention | 6.63±5.01 | 6.33±5.77 | -0.25 (0.46); 0.0 (0.0) | 8.13±5.49 | 11.20±4.60 | -0.25 (0.46); 0.0 (0.0) |
|  | | 3-month follow-up | 6.75±5.18 | 6.33±5.77 | -0.13 (0.35); 0.0 (0.0) | 8.0±5.35 | 11.20±4.60 | -0.38 (0.52); 0.0 (0.0) |
| HK-MoCA (score 0-30, higher is better) | | Baseline | 21.0±0.87 | 20.67±1.21 |  | 20.90±1.37 | 21.20±1.10 |  |
|  | | Post-intervention | 21.63±1.41 | 21.0±1.0 | 0.50 (0.76); 0.0 (0.0) | 21.50±1.69 | 21.40±1.14 | 0.88 (0.64); 0.20 (0.45) |
|  | | 3-month follow-up | 21.63±1.30 | 21.0±1.0 | 0.50 (0.76); 0.0 (0.0) | 21.38±1.51 | 21.60±1.52 | 0.75 (0.46); 0.40 (0.55) |
| TUG (completion time, s, lower is better) | | Baseline | 16.21±5.29 | 16.17±3.82 |  | 16.10±4.60 | 16.18±4.48 |  |
|  | | Post-intervention | 13.88±4.41 | 14.97±0.34 | -1.17 (0.26); -0.4 (0.09) | 15.88±5.38 | 16.13±4.59 | -0.85 (0.75); -0.05 (0.20) |
|  | | 3-month follow-up | 13.94±4.09 | 14.98±0.53 | -1.10 (0.20); -0.03 (0.17) | 16.04±5.70 | 16.07±4.55 | -0.68 (1.16); -0.10 (0.12) |
| CFS-C (score 1–9, lower is better) | | Baseline | 4.11±0.78 | 4.0±0.89 |  | 3.80±1.14 | 3.80±0.84 |  |
|  | | Post-intervention | 3.88±0.64 | 4.0±1.0 | -0.13 (0.35); 0.0 (0.0) | 3.63±1.30 | 3.80±0.84 | -0.25 (0.46); 0.0 (0.0) |
|  | | 3-month follow-up | 4.13±0.84 | 4.0±1.0 | 0.13 (0.35); 0.0 (0.0) | 3.88±1.25 | 3.80±0.84 | 0.0 (0.0); 0.0 (0.0) |

SD, standard deviation; ASMI, Appendicular Skeletal Muscle Mass Index; SPPB, Short Physical Performance Battery; SARC-CalF, Strength, Assistance with Walking, Rise from a Chair, Climb Stairs and Falls; HK-MoCA, Hong Kong version-Montreal Cognitive Assessment; TUG, Timed Up and Go Test; CFS-C, Chinese version of Clinical Frailty Scale; EGRG, exergaming with resistance component group; TRTG, traditional resistance training group
